# Amplifying the Voices of Parents From Underserved Communities in Digital Health for Children With Medical Complexity: Interview Study Among Parents

**DOI:** 10.2196/82317

**Published:** 2026-05-19

**Authors:** Farah Elkourdi, Onur Asan

**Affiliations:** 1 Department of Systems Engineering, Stevens Institute of Technology, 1 Castle Point Terrace, Hoboken, NJ, 07030, United States, 1 2012165514

**Keywords:** children with medical complexity, pediatric care, digital health, mobile health, mHealth, design, care coordination, underserved populations

## Abstract

**Background:**

Children with medical complexity experience multiple chronic conditions that demand intensive, ongoing, and highly coordinated care, often placing a burden on their parents, who serve as primary caregivers. Digital health offers a promising solution for enhancing care coordination, monitoring, and communication. However, its effectiveness depends on it being developed as a user-centered solution that incorporates feedback from parents, who are the primary decision-makers and advocates in their children’s health care. By prioritizing the voices of parents, especially those from underserved communities, during the design and implementation of digital health solutions, these tools can more effectively meet their unique needs. This ensures that digital health solutions are effective in real-world caregiving scenarios.

**Objective:**

This qualitative study explored the experiences of family caregivers of children with medical complexity, with a focus on parents from underserved communities, shedding light on the challenges they face and opportunities for future digital health innovations. Underserved communities in this study are defined as families experiencing structural barriers to accessing specialized care for children with medical complexity, often necessitating additional support from nonprofit or community-based organizations.

**Methods:**

We conducted semistructured interviews with 19 parents of children with medical complexity from underserved communities. All interviews were conducted over Zoom and were audio recorded. We conducted an inductive, reflexive thematic analysis using an iterative codebook to support analytic transparency.

**Results:**

The children in this study had a variety of chronic conditions, each experiencing at least 3 chronic, long-lasting medical conditions. An inductive thematic analysis revealed two broader key themes: (1) virtual care and (2) consumer mobile health (mHealth) apps. The “virtual care” theme focused on the use of remote health care services and communication with health care providers, highlighting parents’ challenges and needs for enhancing virtual care. The “consumer mHealth apps” theme identified needs and challenges in the care management of children with medical complexity that could be addressed through consumer mHealth apps.

**Conclusions:**

This study highlights several insights into the digital health needs of children with medical complexity and their family caregivers. Parents identified a clear and urgent need for telehealth features tailored to better support the unique needs of care for children with medical complexity. Despite the growing adoption of consumer mHealth apps, caregivers reported ongoing challenges, underscoring the necessity for user-centered solutions that are specifically designed with their needs in mind. Future research and development should focus on integrating user feedback to continuously refine and enhance digital health solutions. By addressing these gaps, technology can better empower caregivers and improve the overall health care experience for families of children with medical complexity. Ultimately, this study provides valuable guidance for future digital health innovations to support parents of children with medical complexity from underserved communities.

## Introduction

Children with medical complexity are individuals with multiple chronic conditions—either congenital or acquired—that often involve significant cognitive and/or physical impairments [1,2]. These children typically face functional limitations; rely on life-sustaining medical technologies; and require ongoing, intensive care across multiple body systems [3]. For instance, a child with medical complexity might have a genetic syndrome, a congenital heart defect, cerebral palsy, swallowing difficulties, and a urologic condition [4]. Meeting the needs of children with medical complexity requires highly coordinated, specialized care delivered by a multidisciplinary team of health care professionals [5] along with continuous, collaborative engagement between families and health care providers [6]. Although there is no clear definition of children with medical complexity, they are affected by chronic, often very severe conditions for their entire life, which represents a significant cost for the health care system due to their need for continuous assistance [7]. In our study, we defined “children with medical complexity” as individuals aged 21 years or younger who have at least 3 chronic medical conditions [7].

Parents of children with medical complexity are often the foremost experts on their children’s needs. They shoulder responsibility for both routine and intensive medical care, frequently under unpredictable and high-stakes conditions with few—if any—alternatives to their demanding role [8]. This unpredictability stems from the child’s fragile and often unstable health status [9]. Parents of children with medical complexity perform tasks typically performed by licensed professionals, such as managing life-sustaining technologies, administering medications, and providing complex daily care [10]. As a result, the family caregivers of children with medical complexity report significantly poorer well-being than the general US adult population, including elevated levels of anxiety and impaired sleep health [11].

User-centered digital health solution design incorporating end user input increases the likelihood that digital health solutions will be seen as useful, making it essential to understand patients’ and parents’ access to and interest in such interventions during technology development [12]. Digital health solutions may play an essential role in improving mental and physical health outcomes [13]. The development of digital health technologies represents a rapidly growing area, offering global health care systems opportunities to enhance care and accessibility [14].

This research aimed to elevate the voices of parents of children with medical complexity by exploring their experiences with current digital health tools and gathering their insights into future innovations. As constant caregivers, advocates, and decision-makers, these parents play a central role in managing their children’s complex health care needs and navigating fragmented care systems. By examining how existing technologies support—or fail to support—families of children with medical complexity, this study aimed to identify how digital health can improve care delivery for children with medical complexity and empower families in underserved communities in their caregiving roles. Ultimately, the goal was to identify needs in terms of current digital health solutions and offer actionable recommendations to develop tools that more effectively address the caregiving challenges faced by families of children with medical complexity in underserved communities.

## Methods

### Ethical Considerations

This qualitative study used semistructured interviews to collect data following ethics approval (2023‐006) from the institutional review board of Stevens Institute of Technology. The interview questions focused on identifying challenges and needs in digital health for the care of children with medical complexity (Multimedia Appendix 1). Participation in the study was entirely voluntary, and participants were informed that the data would be reported anonymously. Data access was strictly limited to the study researchers. Each participant was given a US $30 Amazon gift card for completing the interview. We structured our methodology in alignment with the COREQ (Consolidated Criteria for Reporting Qualitative Research) checklist to ensure transparency [15]. The study team received verbal consent from all participants.

### Semistructured Interviews

This qualitative study explored parents’ perspectives on how digital health technology supports them in providing care for children with medical complexity, and this study also has potential to shape the path for future enhanced digital health solutions designed for primary caregivers of children with medical complexity. Participants were recruited through the Statewide Parent Advocacy Network (SPAN) based in New Jersey [16]. SPAN is a community organization for children with special needs and children with medical complexity. It was founded by parents of children with special needs in 1987 to provide support to the families of these children and of children with medical complexity [16]. The staff has supported more than 500 families of children with medical complexity in New Jersey for many years, especially those from underserved communities. We designed a brochure and worked with SPAN members to distribute it to families of children with medical complexity from underserved communities who lacked access to medical services. Participants were family members identified as the primary caregivers for children with medical complexity; most were the children’s parents. We conducted semistructured interviews with primary family caregivers of children with medical complexity from underserved communities between October 2023 and March 2024. In this study, “underserved communities” refers to families who face structural and access-related barriers to specialized care for children with medical complexity, including provider shortages, geographic constraints, fragmented health systems, and challenges navigating services [17]. These barriers are often substantial enough that families rely on nonprofit or community-based organizations (SPAN) for additional support, care coordination, and resource access beyond what is available through the formal health care system. We also used snowball sampling techniques by asking for referrals from the participants. We used the theoretical data saturation approach to determine the final sample size [18]. Data saturation occurs when further interviews do not yield new themes or reveal new subthemes within the existing themes [19,20]. All interviews were conducted over Zoom (Zoom Video Communications) and were audio recorded.

### Data Analysis

An inductive, reflexive thematic analysis was conducted, with a codebook developed iteratively during analysis to support analytic transparency rather than to impose a priori coding structures. We used a constant comparative approach to collect and analyze data simultaneously. Transcripts were analyzed using inductive thematic analysis. The inductive approach focuses on identifying themes as they emerge from the data to understand the needs of children with medical complexity regarding technology preferences and challenges [21]. Inductive thematic analysis ensures that the themes are representative of participant responses and not shaped by a predefined hypothesis [22]. We used a line-by-line coding method [23]. The first author (FE) coded the data and created a codebook, which was then reviewed, refined, and validated by the second author (OA). We identified themes by comparing situations, suggestions, and experiences from the same and different individuals and gradually refined the coding schema [24]. The coding was performed using Microsoft Excel, which included both inductive analysis and participant demographics.

## Results

This study included interviews with 19 family caregivers of children with medical complexity, focusing on parents of children with medical complexity from underserved communities; each interview lasted approximately 45 minutes.

### Demographics

Participant demographics were documented to describe the sample. Table 1 presents a summary of the demographic characteristics of the participating caregivers and their children with medical complexity.

Most participants (15/19, 78.9%) were from New Jersey. However, through snowball sampling, in which existing participants referred others to the study, additional caregivers from California, Alaska, Colorado, and New York were included (4/19, 21.1%). Most (12/19, 63.2%) were African American, with others identifying as Hispanic (3/19, 15.8%) or White (4/19, 21.1%) individuals. Most caregivers were aged between 30 and 49 years. The study included 52.6% (10/19) female and 47.4% (9/19) male participants. All caregivers had 1 child with medical complexity except for one caregiver who had twins with medical complexity. The educational backgrounds of the participants included 10.5% (2/19) with master’s degrees, 73.7% (14/19) with bachelor’s degrees, and 15.8% (3/19) who had completed high school. Finally, most of the children (15/19, 78.9%) were boys. The largest age group was 12 to 14 years.

**Table 1.** Demographic information of the participants.

Participant ID	Age group (y)	Gender	Educational level	Race or ethnicity	State	Relationship to the child	Children with medical complexity, n	Child age (y)	Child gender
P1	40‐49	Male	High school	Black or African American	New Jersey	Father	1	12‐14	Boy
P2	30‐39	Female	Bachelor’s degree	Black or African American	New Jersey	Mother	1	15‐18	Boy
P3	30‐39	Male	Bachelor’s degree	Black or African American	New Jersey	Father	1	12‐14	Boy
P4	30‐39	Male	Bachelor’s degree	Black or African American	Alaska	Father	1	12‐14	Boy
P5	40‐49	Female	Bachelor’s degree	Black or African American	New Jersey	Mother	1	15‐18	Girl
P6	40‐49	Male	Bachelor’s degree	Black or African American	New Jersey	Father	1	6‐11	Boy
P7	40‐49	Male	Bachelor’s degree	Black or African American	New Jersey	Father	1	12‐14	Girl
P8	30‐39	Male	Bachelor’s degree	Black or African American	New Jersey	Uncle	1	6‐11	Boy
P9	30‐39	Male	Master’s degree	Black or African American	Colorado	Father	1	6‐11	Boy
P10	40‐49	Male	Bachelor’s degree	Black or African American	New Jersey	Father	1	12‐14	Boy
P11	30‐39	Male	Bachelor’s degree	Black or African American	New York	Father	1	12‐14	Boy
P12	≥50	Female	Master’s degree	White	New Jersey	Mother	1	6‐11	Boy
P13	40‐49	Female	Bachelor’s degree	Black or African American	California	Mother	1	12‐14	Boy
P14	40‐49	Female	Bachelor’s degree	White	New Jersey	Mother	2; twins	12‐14	Boy and girl
P15	40‐49	Female	Bachelor’s degree	Hispanic or Latino	New Jersey	Mother	1	19‐21	Boy
P16	18‐29	Female	High school	Hispanic or Latino	New Jersey	Mother	1	0‐5	Boy
P17	30‐39	Female	Bachelor’s degree	White	New Jersey	Mother	1	15‐18	Boy
P18	18‐29	Female	High school	Hispanic or Latino	New Jersey	Mother	1	6‐11	Girl
P19	≥50	Female	Bachelor’s degree	White	New Jersey	Mother	1	15‐18	Girl

### Qualitative Analysis

An inductive thematic analysis of the qualitative data revealed 2 primary themes related to current experiences with technology in the care for children with medical complexity and unmet needs that could be addressed through digital solutions. These themes were (1) virtual care and (2) consumer mobile health (mHealth) apps.

#### Virtual Care

The first theme, “virtual care,” refers to any interaction between patients and health care providers occurring remotely, using technology with the aim of facilitating or maximizing the quality and effectiveness of patient care [25]. This theme encompasses subthemes based on parents’ experiences with using virtual care for their children with medical complexity, highlighting both the challenges they encountered and their needs for improving the virtual care process. Table 2 includes the subthemes, their definitions, and supporting quotes.

**Table 2.** Subthemes, definitions, and supporting quotes for the theme of virtual care.

Subtheme	Definition	Quotes
Information and mental overload related to the parental role as mediator	Refers to the overwhelming amount of information and emotional strain while passing on information to health care providers during telehealth sessions	“During the pandemic, it was overwhelming. We had to understand the whole process of how and what my child was going through, because it was at this point that we had to communicate with the doctors more remotely, and we had to pass out information to the service providers.” [P6] “It was just me trying to give all the information to the healthcare provider during the telehealth session, and somehow I kind of felt uncomfortable because I do not know, but I had to do it.” [P5] “You can look at the child, but things are subjective. There are these types of things. How swollen are they? My child’s stomach is hard and painful. How hard is it? There is no clear answer for that. So how do we make sure that the physicians are getting a clear picture of what is happening with the child physically, based on what you see and measure.” [P19]
User-friendly vocabulary	Refers to the need to clearly explain and simplify the medical language so that health information is easy to understand for parents of all levels of medical knowledge	“There are some terminologies I came across that I did not understand. So maybe providing resources where you can actually understand some of the symptoms or what your child is going through. This can be very helpful. We have to carry out personal research. But these are some things that could be included in telehealth services. And it will actually reduce the stress.” [P6]
Support for nonverbal children with medical complexity	Refers to the need to engage, comfort, and support nonverbal children with medical complexity during health care appointments by providing various communication methods based on their abilities and preferences	“My experience is that providers should know how to communicate with a patient with autism who is nonverbal, or how to understand whether they would like to be touched or whether they have struggles waiting like having survey questions to ask the parent before you begin the telehealth session. It really helps to do those pre-screening questions.” [P5] “Some service providers actually do not know how to treat kids who have a hearing impairment, because I know it might be like very tense talking to someone, and the person is finding it hard to hear you.” [P7] “The doctor asked some questions and stuff like that. But facing the screen and answering questions is a bit stressful for my child, and my child can get upset, or just leave the virtual meeting.” [P2]
Child-friendly virtual waiting room	Refers to the need for a digital tool designed to engage, comfort, and entertain children with medical complexity while they wait for their health care appointments	“In terms of the virtual aspects, like sitting, viewing a laptop, and having an appointment, waiting for the doctor. It is kind of intense and stressful for the child.” [P6]

#### Consumer mHealth Apps

The theme “consumer mHealth apps” represents the needs and challenges of parents of children with medical complexity that can be addressed through consumer-focused mHealth features to support their caregiving experience. It explores the features and tools that parents of children with medical complexity need to be developed to manage their caregiving responsibilities effectively. Table 3 presents the subthemes, their definitions, and supporting quotes.

**Table 3.** Subthemes, definitions, and quotes for the theme of consumer mobile health apps.

Subtheme	Definition	Quotes
Digital health notebook	Refers to the need for organizing, tracking, and managing health information in an accessible format	“I write all symptoms down. But it is always kind of hard for me, because, like, in the process of rushing to take my kid to the doctor. Sometimes, I forget to carry the book with me, so I drive back to the house to bring it.” [P10] “I think we need a milestone tracker of where they should be, or where they could be at that age. I think, if it follows them through their milestone and through their ages as they grow and develop, it could be helpful.” [P16]
Medication administration	Refers to the challenges of managing medications for children with medical complexity due to having multiple medications and doses that change frequently	“Change in medication is a big thing, because giving the child certain things at certain times of the day. And suddenly things have changed. So now you have to be able to make sure you are giving it the right time. And there are no other medications that could potentially have a negative effect on the child due to cross interactions.” [P4]
Health technology synchronization	Refers to the need for integrating digital tools and systems related to the care for children with medical complexity to enhance connectivity and user experience	“I think for us, the safety issue is being able to access medical teams that are all on the same page, who can interact, even if they are in different facilities, can communicate with each other effectively.” [P19]
Supporting decision-making	Refers to supporting children with medical complexity in their transition to adult care, enabling them to make informed and confident choices in various situations	“What they really should be learning at the earliest stages is supported decision-making and self-direction supports. Because I was not told. My kids are 12, and I really was not aware of supported decision-making and self-direction until my kids were 9, and I should have been doing this when they were 3.” [P14]

## Discussion

### Principal Findings

Caring for children with medical complexity involves navigating an extensive network of health care providers—primary care clinicians, specialists, and nurses—across multiple settings, including the home, school, clinics, and hospitals. This fragmentation increases the risk of care gaps over time [26-28]. Prior studies have shown that parents of children with medical complexity face multiple barriers to communication and information accessibility, including difficulties scheduling appointments; coordinating among health care providers; and obtaining clear, actionable health information [29-32]. Many are also tasked with administering complex medication regimens without formal training or having children with medical complexity–specific tools to ensure accuracy [33]. These challenges are even more pronounced for families in underserved communities.

Underserved populations who lack access to proper health care infrastructure are rapidly increasing their adoption of digital health tools to address gaps in care by overcoming barriers such as language and geography [34,35]. A previous study shows that further research is needed to evaluate the effectiveness of mHealth and virtual care and their impact on health care delivery in underserved communities [36]. In addition, digital health tools for the care for children with medical complexity should be shaped by the voices and experiences of families on the front lines of care to build systems that work for children with medical complexity [37]. A unique aspect of this study is its focus on children with medical complexity in underserved communities as a first step, revealing how caregiving challenges intersect with the realities of digital health use with the goal of advancing user-centered digital health solutions for parents of children with medical complexity.

Telehealth has been suggested as a possible way to address health care disparities among underserved urban populations who are often unable to access health care in a timely manner due to low physician-to-population ratios and limited specialty care [38]. Accordingly, telehealth is viewed as a strategy for improving access to care for rural and underserved populations by connecting them with primary care providers, specialists, and mental health services [39]. While virtual care offers a promising avenue for overcoming geographic and resource-related barriers [40], our findings demonstrate that it can also shift significant responsibility onto caregivers. Parents described the mental and emotional overload of acting as the primary information conduit between child and health care provider, particularly during remote visits.

Therefore, health literacy is essential for parents of children with medical complexity to make informed decisions and manage their children’s specialized care needs [41,42]. Low parental health literacy has been associated with limited health knowledge and poorer child health outcomes [43,44]. Consistent with this, our participants emphasized that integrating user-friendly vocabulary and parent-centered resources within telehealth platforms can reduce the cognitive burden on parents by enabling them to interpret information immediately while communicating with health care providers via telehealth and actively participate in their children’s care.

While parental understanding supports a child’s care, providers also need to adopt strategies that allow them to communicate effectively with nonverbal children with medical complexity during telehealth appointments. A previous study reported that medical providers’ communication strategies often failed to address the needs of nonverbal children with medical complexity, and that strategies such as warmly greeting patients and introducing themselves were sometimes skipped based on the false assumption that these patients could not engage in meaningful communication, which in turn diminished trust between patients and medical providers [45]. This supports our findings that telehealth tools need to be designed as child friendly and support nonverbal children with medical complexity to facilitate communication between patients and health care providers. These considerations were identified as critical for developing an inclusive telehealth tool and are often overlooked in current platforms.

In addition, mHealth is a type of digital health that refers to the use of mobile technologies to deliver health services. It is particularly valuable for improving health outcomes in patients with chronic conditions [12]. Families of children with medical complexity commonly struggle to accurately recall symptoms, clinical events, or over-the-counter medication use, highlighting the need for mHealth tools to document health events outside clinical settings [46]. Regarding caregiver needs, our participants sought mHealth solutions to reduce daily burden and empower decision-making. They envisioned tools consolidating digital health notebooks and medication management. These findings align with those of previous research on the potential of mHealth to empower patients through personalized, timely support [47,48] and integrate digital tools across home, health care, and school systems [49-52]. The desire for synchronized health technology highlights a critical gap in current systems as caregivers of children with medical complexity must repeatedly communicate the same information across providers, increasing workload and the risk of errors.

In addition to supporting day-to-day care, participants emphasized the need for digital technologies that facilitate decision-making for children with medical complexity as they transition to adult care. Few studies have examined the effectiveness of digital health interventions to assist adolescents and young adults with chronic conditions in facilitating self-management and transitioning to adult care [53,54]. There is an interest in using mHealth tools to facilitate the transition to adult care, but early co-design with users and collaboration with health care providers are needed to address varying preferences and needs [53].

**Figure 1.**
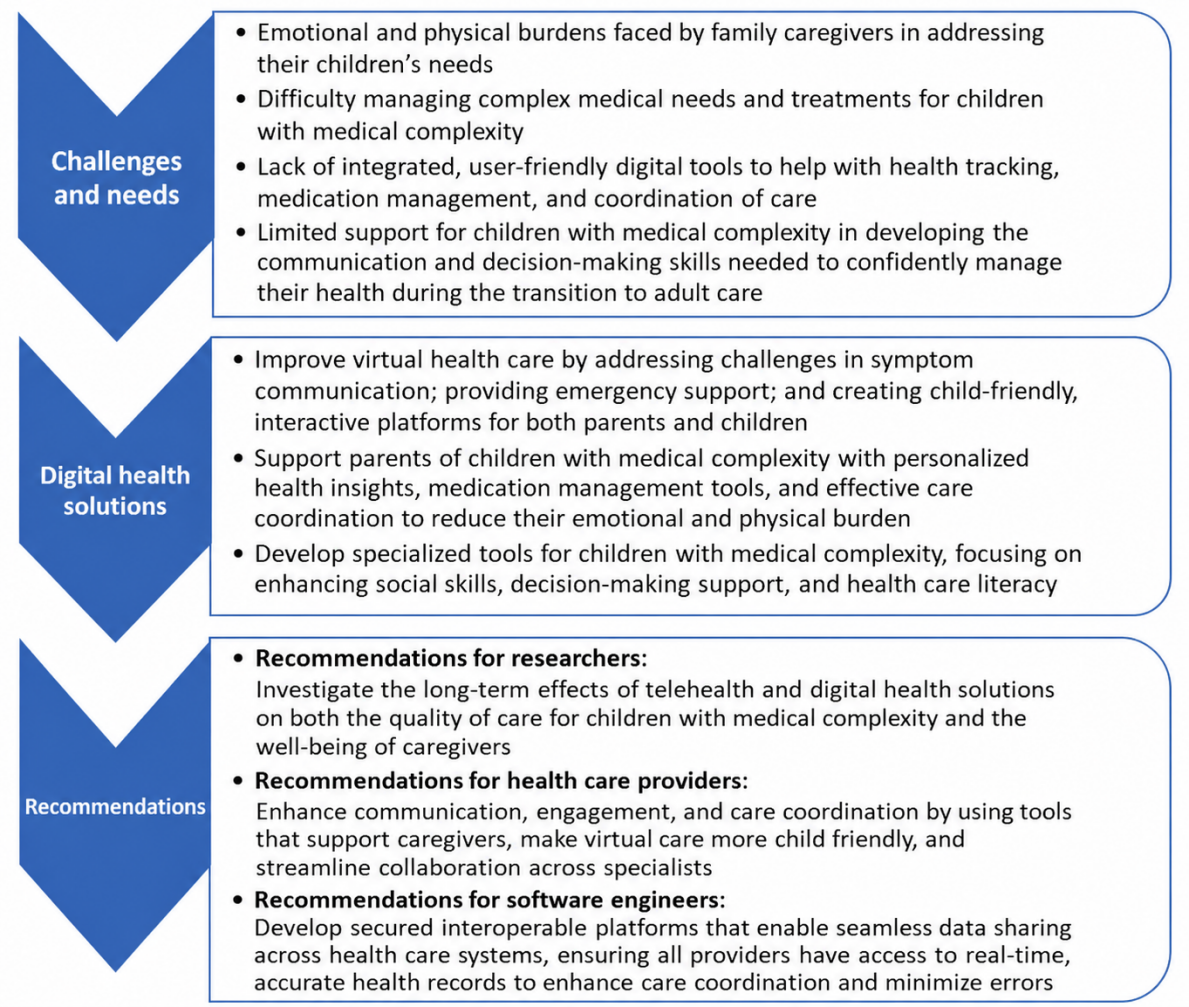
Challenges, solutions, and recommendations for enhancing care for children with medical complexity through technological and supportive interventions.

Figure 1 summarizes the key challenges that parents of children with medical complexity face—managing complex needs, accessing specialized resources, and coping with emotional and physical burdens—that emerged from our findings. We also developed and listed targeted solutions and recommendations for researchers, health care providers, and software engineers to develop effective, user-friendly systems that better support families.

As part of the user-centered design approach, we developed key digital health functionalities to address participants’ needs and bridge gaps in the care for children with medical complexity (Table 4). Effective mHealth tools begin with a user-centered design approach that engages end users throughout development to ensure functionality, usability, and relevance [55-57]. Guided by this principle, we assessed the needs of parents of children with medical complexity and translated them into recommended mHealth platforms, including a description of their functionalities to inform the development of actionable software solutions. Technology developers must also ensure compliance with HIPAA (Health Insurance Portability and Accountability Act) alongside robust security, reliability, and usability in collaboration with health care leaders and policymakers [58-60]. In addition, expanding access to digital health tools in underserved communities requires investment in telehealth infrastructure, mobile-friendly screening tools, and community outreach programs that educate caregivers on how to use these technologies effectively [61].

**Table 4.** Technological solutions to support caregivers in underserved communities.

Subtheme	Recommendation	Description
Information and mental overload related to the parental role as mediator	Centralized child-specific care app	A personalized platform tailored to each child’s unique needs, providing a database of treatments, technologies, and resources for mobility, speech, and feeding; contact information for specialists and facilities; and video tutorials and step-by-step guides on managing medical devices, administering medications, and responding to emergencies at home
Medication administration	Medication and device management tool	An app with features providing barcode scanning for medications; automated reminders for dosage and allergy tracking; integration with medical devices to monitor use and performance; medication interaction checker (the app cross-references medications and alerts parents if there are potential interactions or adverse effects); and notifications to reorder critical medical supplies, with direct links to pharmacies or online retailers
Support for nonverbal children with medical complexity, child-friendly virtual waiting room, and information and mental overload related to the parental role as mediator	Virtual assistant and follow-up tracker	A virtual assistant app that sets follow-up reminders after treatment; logs symptoms automatically during video consultations; provides summaries of past interactions with physicians; analyzes logged symptoms over time and provides reports, highlighting patterns or triggers for review with health care providers; automatically translates medical terminology into simple, parent-friendly language during telehealth interactions; integrates a pre–telehealth visit form to identify the child’s communication preferences, including visual aids, text-based prompts, captioning, or other alternative input methods, to support nonverbal children during telehealth visits; and provides an interactive virtual waiting room with games and calming visuals to reduce children’s stress while waiting for appointments
Information and mental overload related to the parental role as mediator	Medical team coordination tool	A shared platform for health care providers that links professionals from different facilities; offers real-time updates on the child’s medical condition; allows caregivers to monitor progress remotely; provides shared medical records and care plans; allows for easy access to specialists in cases of emergency; features a one-tap emergency alert button that notifies all members of the health care team and shares the latest health data; and includes a comprehensive profile that can be shared with new health care providers, including past treatments, medications, and allergies
Supporting decision-making and information and mental overload related to the parental role as mediator	Enhanced appointment and scheduling system	A system for booking appointments that integrates with personal reminders and health care databases Parental controls to allow older children to schedule appointments independently Ability to set appointments and notifications in the caregiver’s preferred language to accommodate diverse backgrounds
Information and mental overload related to the parental role as mediator	Stress relief features for parents	A caregiver-focused app with emotional support resources such as online counseling; guided journaling to reduce the mental load of caregiving; sleep and stress management techniques; and quick, step-by-step instructions for handling medical emergencies, with links to emergency contacts or services
Health technology synchronization	Smart integration with existing technology	Compatibility with devices such as Alexa or Google Assistant for reminders and notifications A mobile-friendly design that integrates seamlessly with smart home ecosystems Hands-free voice commands to log symptoms, check schedules, or receive reminders A shared care dashboard that syncs updates across caregivers and medical teams

### Strengths and Limitations

To our knowledge, this is the first study to examine the needs and challenges related to digital health technology among parents of children with medical complexity in underserved communities. A key strength of this study is the inclusion of a relatively large sample of caregivers from underserved communities, a group that is frequently underrepresented in qualitative research on children with medical complexity. Although participants were recruited from underserved communities, the relatively high educational attainment among caregivers in our sample may limit the generalizability of the findings to caregivers with lower levels of education. Another limitation of this study is its reliance on Zoom interviews, which may have excluded participants with limited access to technology or who were uncomfortable with digital platforms. This could result in a sample that does not fully capture the experiences of parents who might prefer face-to-face communication.

### Conclusions

Our study reinforces that parents in underserved communities are not resistant to digital health; rather, they are eager for solutions that are trustworthy, accessible, and tailored to the care of children with medical complexity. By addressing the intersecting barriers of medical complexity and systemic inequity, health care systems and technology developers can design digital tools that meaningfully enhance care coordination, reduce caregiver burden, and improve health outcomes. Future work should focus on co-design approaches with families of children with medical complexity, evaluation of tailored interventions, and longitudinal research to examine impacts on care transitions and health care use.

## Supplementary material

10.2196/82317 Multimedia Appendix 1 Interview guide.

10.2196/82317 Checklist 1 COREQ checklist.

## Abbreviations

COREQ: Consolidated Criteria for Reporting Qualitative Research
HIPAA: Health Insurance Portability and Accountability Act
mHealth: mobile health
SPAN: Statewide Parent Advocacy Network

## References

[1] Cohen E, Kuo DZ, Agrawal R (2011). Children with medical complexity: an emerging population for clinical and research initiatives. Pediatrics.

[2] Berry JG, Hall M, Cohen E, O’Neill M, Feudtner C (2015). Ways to identify children with medical complexity and the importance of why. J Pediatr.

[3] Rogers J, Reed MP, Blaine K, Manning H (2021). Children with medical complexity: a concept analysis. Nurs Forum.

[4] Kuo DZ, Houtrow AJ, Council on Children With Disabilities (2016). Recognition and management of medical complexity. Pediatrics.

[5] Asan O, Elkourdi F, Super I, Rezaeian O, Percy S, Clouser K (2025). Children with medical complexity care journey during COVID-19 from providers perspective: a qualitative study. BMC Health Serv Res.

[6] Williams LJ, Waller K, Chenoweth RP, Ersig AL (2021). Stakeholder perspectives: communication, care coordination, and transitions in care for children with medical complexity. J Spec Pediatr Nurs.

[7] Asan O, Super I, Percy S, Clouser KN (2025). The effect of COVID-19 on health care utilization among children with medical complexity: retrospective chart review study. JMIR Pediatr Parent.

[8] Mackay L, Dewan T, Asaad L (2026). The health and well-being of children with medical complexity and their parents’ when admitted to inpatient care units: a scoping review. J Child Health Care.

[9] Rennick JE, St-Sauveur I, Knox AM, Ruddy M (2019). Exploring the experiences of parent caregivers of children with chronic medical complexity during pediatric intensive care unit hospitalization: an interpretive descriptive study. BMC Pediatr.

[10] Murphy NA, Darro N (2021). Parents of children with medical complexity are essential health care personnel. Pediatrics.

[11] McLachlan LM, Engster S, Winger JG (2024). Self-reported well-being of family caregivers of children with medical complexity. Acad Pediatr.

[12] Heneghan MB, Hussain T, Barrera L (2021). Access to technology and preferences for an mHealth intervention to promote medication adherence in pediatric acute lymphoblastic leukemia: approach leveraging behavior change techniques. J Med Internet Res.

[13] Ramsey WA, Heidelberg RE, Gilbert AM, Heneghan MB, Badawy SM, Alberts NM (2020). eHealth and mHealth interventions in pediatric cancer: a systematic review of interventions across the cancer continuum. Psychooncology.

[14] Matricardi PM, Dramburg S, Alvarez-Perea A (2020). The role of mobile health technologies in allergy care: an EAACI position paper. Allergy.

[15] Tong A, Sainsbury P, Craig J (2007). Consolidated criteria for reporting qualitative research (COREQ): a 32-item checklist for interviews and focus groups. Int J Qual Health Care.

[16] SPAN’s history & mission. SPAN.

[17] Crossley S, Baybutt M (2024). Access to healthcare: underserved communities. Int J Health Promot Educ.

[18] Van Orne J (2022). Care coordination for children with medical complexity and caregiver empowerment in the process: A literature review. J Spec Pediatr Nurs.

[19] Nelson J (2017). Using conceptual depth criteria: addressing the challenge of reaching saturation in qualitative research. Qual Res.

[20] Charmaz K (2006). Constructing Grounded Theory: A Practical Guide through Qualitative Analysis.

[21] Naeem M, Ozuem W, Howell K, Ranfagni S (2023). A step-by-step process of thematic analysis to develop a conceptual model in qualitative research. Int J Qual Methods.

[22] Novak M, Drummond K, Kumar A (2022). Healthcare professionals’ experiences with education in short term medical missions: an inductive thematic analysis. BMC Public Health.

[23] Williams M, Moser T (2019). The art of coding and thematic exploration in qualitative research. Int Manag Rev.

[24] Mishra S, Dey AK (2022). Understanding and identifying ‘themes’ in qualitative case study research. South Asian J Bus Manag Cases.

[25] Mistry SK, Shaw M, Raffan F (2022). Inequity in access and delivery of virtual care interventions: a scoping review. Int J Environ Res Public Health.

[26] Adams S, Beatty M, Moore C (2021). Perspectives on team communication challenges in caring for children with medical complexity. BMC Health Serv Res.

[27] Quigley L, Lacombe-Duncan A, Adams S, Hepburn CM, Cohen E (2014). A qualitative analysis of information sharing for children with medical complexity within and across health care organizations. BMC Health Serv Res.

[28] Ranade-Kharkar P, Weir C, Norlin C (2017). Information needs of physicians, care coordinators, and families to support care coordination of children and youth with special health care needs (CYSHCN). J Am Med Inform Assoc.

[29] Schnell JL, Tager JB, Kenney AE (2025). Impact of systems of care on emotional well-being of primary family caregivers of children with medical complexity. Matern Child Health J.

[30] Mesman GR, Kuo DZ, Carroll JL, Ward WL (2013). The impact of technology dependence on children and their families. J Pediatr Health Care.

[31] Elkourdi F, Asan O Empowering children with medical complexity: the role of technology in bridging education and healthcare. https://researchwith.stevens.edu/en/publications/empowering-children-with-medical-complexity-the-role-of-technolog/.

[32] Elkourdi F, Asan O (2025). Health IT in mental health care: supporting children with medical complexity and their parents. Proc Hum Factors Ergon Soc Annu Meet.

[33] Jolliff A, Coller RJ, Kearney H (2024). An mHealth design to promote medication safety in children with medical complexity. Appl Clin Inform.

[34] Cruz S, Lu C, Ulloa M, Redding A, Hester J, Jacobs M (2024). Perceptions of wearable health tools post the COVID-19 emergency in low-income Latin communities: qualitative study. JMIR Mhealth Uhealth.

[35] Dabbs ADV, Myers BA, Mc Curry KR (2009). User-centered design and interactive health technologies for patients. Comput Inform Nurs.

[36] Anderson-Lewis C, Darville G, Mercado RE, Howell S, Di Maggio S (2018). mHealth technology use and implications in historically underserved and minority populations in the United States: systematic literature review. JMIR Mhealth Uhealth.

[37] Agrawal R, Stille C (2018). Building systems that work for children with complex health care needs: editor’s note. Pediatrics.

[38] Montague E, Perchonok J (2012). Health and wellness technology use by historically underserved health consumers: systematic review. J Med Internet Res.

[39] Park J, Erikson C, Han X, Iyer P (2018). Are state telehealth policies associated with the use of telehealth services among underserved populations?. Health Aff (Millwood).

[40] Marcin JP, Ellis J, Mawis R, Nagrampa E, Nesbitt TS, Dimand RJ (2004). Using telemedicine to provide pediatric subspecialty care to children with special health care needs in an underserved rural community. Pediatrics.

[41] Desmarais AV, Kevill K, Glick AF (2024). The complex impact of health literacy among parents of children with medical complexity. Hosp Pediatr.

[42] Goodwin EJ, Zaniletti I, Solano J (2024). Parental health literacy and acute care utilization in children with medical complexity. Hosp Pediatr.

[43] Zaidman EA, Scott KM, Hahn D, Bennett P, Caldwell PH (2023). Impact of parental health literacy on the health outcomes of children with chronic disease globally: a systematic review. J Paediatr Child Health.

[44] Mörelius E, Robinson S, Arabiat D, Whitehead L (2021). Digital interventions to improve health literacy among parents of children aged 0 to 12 years with a health condition: systematic review. J Med Internet Res.

[45] Schnaith A, Schifsky J, Schifsky T, Pitt MB (2021). Communication strategies for patients who are nonverbal. Pediatrics.

[46] Sezgin E, Noritz G, Elek A (2020). Capturing at-home health and care information for children with medical complexity using voice interactive technologies: multi-stakeholder viewpoint. J Med Internet Res.

[47] Korpershoek YJ, Hermsen S, Schoonhoven L, Schuurmans MJ, Trappenburg JC (2020). User-centered design of a mobile health intervention to enhance exacerbation-related self-management in patients with chronic obstructive pulmonary disease (Copilot): mixed methods study. J Med Internet Res.

[48] Molina-Recio G, Molina-Luque R, Jiménez-García AM, Ventura-Puertos PE, Hernández-Reyes A, Romero-Saldaña M (2020). Proposal for the user-centered design approach for health apps based on successful experiences: integrative review. JMIR Mhealth Uhealth.

[49] Karataş N, Kaya A, İşler Dalgıç A (2022). The effectiveness of user-focused mobile health applications in paediatric chronic disease management: a systematic review. J Pediatr Nurs.

[50] Elkourdi F, Asan O (2025). Community caregivers' perspectives on Health IT use for children with medical complexity: qualitative interview study. JMIR Pediatr Parent.

[51] Elkourdi F, Asan O Healthcare providers’ recommended technology features to enhance care for children with medical complexity: a qualitative study.

[52] Werner NE, Morgen M, Kooiman S (2024). Effectiveness of a mobile app (Meds@HOME) to improve medication safety for children with medical complexity: protocol for a randomized controlled trial. JMIR Res Protoc.

[53] Li Z, Lu F, Wu J (2024). Usability and effectiveness of eHealth and mHealth interventions that support self-management and health care transition in adolescents and young adults with chronic disease: systematic review. J Med Internet Res.

[54] Virella Pérez YI, Medlow S, Ho J, Steinbeck K (2019). Mobile and web-based apps that support self-management and transition in young people with chronic illness: systematic review. J Med Internet Res.

[55] Schnall R, Rojas M, Bakken S (2016). A user-centered model for designing consumer mobile health (mHealth) applications (apps). J Biomed Inform.

[56] Bonet-Olivencia S, Carrillo-Leal J, Rao A, Sasangohar F (2024). User-centered design of a diabetes self-management tool for underserved populations. J Diabetes Sci Technol.

[57] Griffin L, Lee D, Jaisle A (2019). Creating an mHealth app for colorectal cancer screening: user-centered design approach. JMIR Hum Factors.

[58] Elkourdi F, Wei C, Xiao L, Yu Z, Asan O (2024). Exploring current practices and challenges of HIPAA compliance in software engineering: scoping review. IEEE Open J Syst Eng.

[59] Galetsi P, Katsaliaki K, Kumar S (2022). Assessing technology innovation of mobile health apps for medical care providers. IEEE Trans Eng Manage.

[60] Elkourdi F, Asan O, Mansouri M The unintended effects of medical software on clinical decisions and patient safety: a system viewpoint. https://personales.upv.es/thinkmind/dl/conferences/icons/icons_2023/icons_2023_1_30_40020.pdf.

[61] Kadam SJ, Goel M (2025). Bridging the gap: autism spectrum disorder in children in the United States and worldwide: a narrative review. Clin Exp Pediatr.

