# Supplementary material for: Amplifying the Voices of Parents From Underserved Communities in Digital Health for Children With Medical Complexity: Interview Study Among Parents
Source: JMIR Pediatr Parent. 2026 May 19;9:e82317. doi: 10.2196/82317 (PMC13227322; doi:10.2196/82317)
Supplement: Multimedia Appendix 1 [file pediatrics-v9-e82317-s001.docx]

**Interview Guide**

**Family Caregiver/ Parent Interviews**

**Section 1. General Questions**

1. Can you describe your child's medical condition and care needs?
2. In general, have you encountered any gaps or challenges in your child's medical care or support services? If so, could you please share some specific examples?

**Section 2. Telehealth Questions**

1. How was your overall experience with virtual care (telehealth etc.) in CMC care?
2. How effective has telehealth been in addressing the specific medical needs of your child with medical complexity?
3. If we want to integrate features in telehealth to better address children with medical complexity needs, what might it be?

**Section 3. Technology Questions**

1. Are you aware of any technology that support CMC care and parents, Is it useful? Why or why not?
2. Do you have any suggested modifications you wish to add to the digital solutions that you are currently using?
3. How can software/ technology help to improve safety and prevent errors for children with medical complexity?
4. What were the challenges or limitations in using technology to support your child’s care needs during the pandemic?
